# HMGB1 overexpression promotes a malignant phenotype and radioresistance in ESCC

**DOI:** 10.7150/jca.73761

**Published:** 2022-05-21

**Authors:** Jing Dong, Xueyuan Zhang, Xingyu Du, Naiyi Zou, Wenbin Shen, Ming Ma, Yaojie Wang, Shuchai Zhu

**Affiliations:** 1 Department of Radiation Oncology, The Fourth Hospital of Hebei Medical University, Shijiazhuang, Hebei, P. R. China; 2 Department of Clinical Laboratory, The Fourth Hospital of Hebei Medical University, Shijiazhuang, Hebei, P. R. China; 3 Research Center, The Fourth Hospital of Hebei Medical University, Shijiazhuang, Hebei, P. R. China

**Keywords:** ESCC, HMGB1, malignant phenotype, radioresistance, γ-H2AX

## Abstract

Esophageal cancer is a common malignant disease that is generally treated with radiotherapy. High mobility group box 1 (HMGB1) plays an essential role in tumor cell proliferation, migration, and cell cycle progression. Here, we aimed to clarify the effects of HMGB1 on radioresistance in esophageal squamous cell carcinoma (ESCC) cell lines and patient survival. We performed immunohistochemistry for HMGB1 in biopsy samples of 39 stage I-III ESCC patients grouped by HMGB1 expression status. Then, 1‐, 3‐, 5-, and 10‐year overall survival outcomes were calculated by Kaplan-Meier survival analysis. The cellular localization of HMGB1 was examined before and after irradiation by Immunofluorescence staining. Stable cell lines (KYSE30 and KYSE510) with differential HMGB1 expression were constructed using lentiviruses. Furthermore, we examined phosphorylated histone H2AX (γ-H2AX) expression in both HMGB1 overexpression and negative control groups by western blotting. HMGB1-negative expression was associated with superior ESCC patient 10-year survival (*P*=0.016). HMGB1 overexpression promoted cell migration, proliferation, and radioresistance and mitigated cell cycle arrest at the G0/G1 phase induced by irradiation. This demonstrates that HMGB1-positive expression is correlated with unfavorable clinical outcomes, and HMGB1 overexpression may promote the malignant phenotype of ESCC cells and induce radioresistance by regulating cell cycle distribution in ESCC.

## Introduction

Esophageal cancer (EC) is one of the gastrointestinal tumors and is the eighth most common malignancy worldwide 1, 2. Radiotherapy is an important modality for treating EC 3. The continuous advances in radiotherapy equipment and technology have improved the prognosis of EC patients. However, anti-tumor efficacy remains suboptimal in part because of radioresistance 4. Radioresistance in patients has become a focal point of EC research, especially in esophageal squamous cell carcinoma (ESCC), because it is the globally predominant pathological type of EC 5. High mobility group box 1 (HMGB1) is a non-histone chromatin-binding protein in eukaryotes and is an essential protein for life. HMGB1 is involved in the modulation of cancer cell proliferation, invasion, and metastasis 6, 7. HMGB1 can affect the efficacy of radiotherapy and has an impact on the survival of cancer patients. Jiao et al. proposed that by targeting the HMGB1-RB axis, SEPT9 methylation promoted tumorigenesis and radioresistance of cervical cancer 8, and under chemoradiotherapy or hypoxia, HMGB1 can be translocated 9. In addition, high levels of HMGB1 protein expression are reportedly associated with poor prognosis of colorectal cancer patients 10.

Radiotherapy not only mediates a therapeutic response by inducing DNA double-strand breaks (DSBs) but also has immunomodulatory effects. Irradiation can induce translocation of HMGB1 to the cytoplasm, and it can regulate cell proliferation and migration by binding to its high‐affinity receptor. Intracellular HMGB1 regulates DNA repair systems and autophagy in tumor cells to induce radioresistance. Extracellular HMGB1 has a complex role in the radiation-related immune response. It not only stimulates the anti-tumor immune response by promoting the recognition of dying tumor cells but also participates in maintaining immunosuppression 11, 12.

Zhang et al. proposed that X-ray irradiation induces dying cells to release HMGB1 promoting CD133- pancreatic cancer cells to maintain stemness. Both *in vitro* and PDX models showed that inhibiting HMGB1 expression in irradiated, dying cells could attenuate the dedifferentiation stimulation effect on C133- pancreatic cancer cells. X-ray irradiation-induced CD133- pancreatic cancer cells to differentiate into the cancer stem cell (CSC) phenotype, and preventing CSC enrichment and pancreatic carcinoma relapse by inhibiting HMGB1 may be a useful strategy 13. Similarly, silencing HMGB1 expression could reportedly attenuate the stimulating effects on the migration of irradiated, dying cells, promotion of cancer cell migration via paracrine mechanisms, and enhanced epithelial-mesenchymal transition (EMT) and PI3K/pAkt signaling. During radiotherapy, the dying cells could activate paracrine signaling to facilitate the migration of surviving tumor cells. From these data, Chen et al. suggested that inhibiting HMGB1 could help prevent pancreatic carcinoma relapse and metastasis 14.

It is confirmed that knockdown of HMGB1 could decrease the proliferation and invasive ability of non-small cell lung cancer cells while also increasing the radiosensitivity of the cells 15. Shrivastava et al. showed a positive correlation between high levels of HMGB1 and radioresistance in bladder cancer cell lines. After HMGB1 knockdown, the radiosensitivity of bladder cancer cells was increased. They found that HMGB1 is possibly a crucial gene in these cells because its loss was related to higher radiation-induced cellular DNA damage levels. These data highlight the potential of HMGB1 to be a radiation marker, and the capability to enhance radiation efficacy 16. Similarly, Ayoub et al. proposed that HMGB1 might play a role in radioresistance of bladder cancer by promoting pro-tumor immune mechanisms, such as reducing the frequency of pro-tumor myeloid-derived suppressor cells and tumor-associated macrophages 17.

In EC, Matsubara et al. proposed that high levels of HMGB1 in tumor cells or plasma could play a key role in the malignant potential of ESCC 18. Takahata suggested that a high HMGB1 expression level was a risk factor for severe postoperative pulmonary complications in EC patients 19. HMGB1 may serve as a new therapeutic target or clinical biomarker for metastatic ESCC 20. It was confirmed that downregulating HMGB1 expression could promote radiosensitivity in ESCC both *in vitro* and *in vivo* 21, 22. Moreover, activating a DNA damage checkpoint response after irradiation was the chief cause of HMGB1-induced radioresistance 23.

It was previously reported that HMGB1 expression levels were significantly associated with ESCC patient survival 18. The relationship between knocking down HMGB1 expression in ESCC cells and radiosensitivity has been studied, which reported that knockdown HMGB1 expression in ECA109 and TE13 cell lines increased their radiosensitivity 24. However, there are few studies focused on HMGB1 overexpression. Therefore, it is necessary to research to verify the relationship between HMGB1 overexpression and radioresistance in ESCC by selecting appropriate cell lines. Better understanding the effect of HMGB1 on radiosensitivity and identifying radioresistance in ESCC patients may provide novel insights. Hence, our study aimed to explore the effect of HMGB1 overexpression on cell proliferation, cell cycle progression, and radiosensitivity of ESCC cell lines and to provide a theoretical basis for the treatment of ESCC.

## Material and Methods

### Immunohistochemistry (IHC)

This study was approved by the Ethics Committee of the Fourth Hospital of Hebei Medical University (IRB approval ID: 2020KY184). All patients gave verbal informed consent to participate in the study. Patient inclusion criteria were 1. age ≥18 years; 2. pathological diagnosis of first primary ESCC; 3. not receiving any treatment at the time of biopsy sample collection; 4. having received radiotherapy alone; 5. complete general and pathological data; and 6. patient informed consent. From January 2008 to November 2013, pretreatment biopsy specimens from 39 stage I-III ESCC patients were retrospectively collected. The patients were treated with radiotherapy alone at the Fourth Hospital of Hebei Medical University. A standard IHC staining procedure was followed. The IHC staining was carried out as described 24, 25. The localization of HMGB1 cells was observed microscopically, and the IHC results of the sections were assessed by light microscopy by two chief pathologists under independent double-blind conditions. Referring to the assessment method proposed by Soumaoro et al. 26, the entire field of view of the tumor tissue, the intensity of positive cell coloration, and the percentage of positive cells were assessed when the sections were observed under the microscope. The intensity of positive cell coloration was scored as 0, 1, 2, and 3, corresponding to unstained, yellowish, brownish, and tan. The percentage of positive cells was scored as 0, l, 2, 3, and 4, corresponding to 0%, 1-25%, 26-50%, 51-75%, and 76-100% of positive cells in that order. The two scores were summed for evaluation, and the resultant tumor tissue specimens with scores of 0-2 were considered to have negative HMGB1 protein expression Scores greater than or equal to 3 were judged to have positive HMGB1 protein expression.

### Cell culture

Ethical approval for this study was obtained from the Ethics Committee of The Fourth Hospital of Hebei Medical University. The KYSE180, ECA109, TE1, KYSE150, KYSE510, and KYSE30 ESCC lines were obtained from the Research Center of The Fourth Hospital of Hebei Medical University (Shijiazhuang, China). In addition, the cells were cultured in RPMI 1640 medium (Gibco, Beijing, China), which contained 100 U/mL penicillin-streptomycin solutions (Solarbio, Beijing, China) and 10% fetal bovine serum (FBS, Cellmax, Lanzhou, China). The cultures were stored in an incubator (5% CO_2_ and saturated humidity) at 37°C.

### Immunofluorescence staining

Exponentially proliferating ESCC KYSE30 and KYSE510 cells were seeded in 24-well plates for culture for 12 h. The cells of the 6 Gy groups were irradiated by 6 MV X-rays (dosage rate of 2 Gy/min) and generated by a linear accelerator (Siemens, Buffalo Grove, IL, USA). The controls were not treated. The cells were cultured consecutively for two h and then fixed in 4% paraformaldehyde at room temperature for 20 min. After being rinsed three times with phosphate buffer saline (PBS, Gibco, Beijing, China), the cells were permeabilized with 0.2% Triton X-100 (Vetec, Wuxi, China) in PBS for 10 min and blocked with 10% goat serum (Solarbio, Beijing, China) for 30 min. Then cells underwent incubation with antibody anti-HMGB1 (dilution 1:500; cat. no. 10829-1-AP; Proteintech) at 4°C for 12 h. After three rinses with PBS, the samples underwent incubation for one h with green fluorescent Goat Anti-Rabbit IgG (dilution 1:200; cat. no. SA00013-3; Proteintech), protected from light at room temperature. Following the staining of cell nuclei with DAPI (Solarbio, Beijing, China), cells were viewed under an ECLIPSE Ti2 confocal laser scanning microscope (Nikon, Tokyo, Japan).

### Cell transfection

The HMGB1 overexpression lentiviruses were obtained from Gene Chem Co., Ltd (Shanghai, China). The cells were then cultured until they reached 50% confluence in 6-well plates. Next, the lentivirus with HMGB1 overexpression and the negative control (NC) lentiviral particles were added to the cultures for 12 h (MOI=10). Another 36 h later, transfection efficiency was observed under the fluorescence microscope (Nikon, Tokyo, Japan). The cells were then selected and cultured with puromycin (1 μg/mL) for two weeks.

### Real-time quantitative polymerase chain reaction (RT-qPCR)

RNA extraction and RT-qPCR were performed as Zhang et al. described (24). And the primer sequences for RT-qPCR included the following:

HMGB1, F: 5'-AATACGAAAAGGATATTGCT-3', R: 5'-GCGCTAGAACCAACTTAT-3'; GAPDH, F: 5'-CGCTGAGTACGTCGTGGAGTC-3', R: 5'-GCTGATGATCTTGAGGCTGTTGTC-3'.

### Western blotting

Lysis of cells was conducted with radioimmunoprecipitation buffer (Solarbio, Beijing, China), which contained 1% phenylmethanesulfonyl fluoride (Solarbio, Beijing, China) and 1% phosphatase inhibitor (MCE, New Jersey, USA). The samples were centrifuged at 8000 rpm at 4°C for 15 min, and then the supernatant was collected. The concentration of proteins was detected by a BCA protein assay kit (Solarbio, Beijing, China), and Laemmli buffer was added to prepare protein samples. The solution was heated for 5 min at 100°C, followed by analysis with sodium dodecyl sulfate-polyacrylamide gel electrophoresis. Subsequently, proteins were transferred to polyvinylidene fluoride (PVDF) membranes. After being blocked with 5% nonfat dry milk for one h, PVDF membranes underwent incubation employing specific primary antibodies at 4°C overnight. Following three rinses with TBS containing 0.1% Tween 20 (TBST) at room temperature, incubation of membranes was performed by utilizing second antibodies for one h. After three more rinses with TBST, the membranes were imaged using the Odyssey system (LI-COR Biosciences, Lincoln, NE, USA). In this process, the antibodies used were as follows: anti-HMGB1 (dilution 1:1,000; cat. no. 10829-1-AP; Proteintech), anti-γ-H2AX (dilution 1:1,000; cat. no. ab81299; Abcam), anti-β-actin (dilution 1:5,000; cat. no. 60008-1-Ig; Proteintech), Goat pAb to Rb (dilution 1:1,0000; cat. no. ab216777; Abcam) and Goat pAb to Ms (dilution 1:1,0000; cat. no. ab216772; Abcam). Image J software was used to quantify autoradiographic bands, then normalize for β-actin levels.

### Cell proliferation assay

The cell suspension was seeded into 96‐well plates (100 μL/well; five wells per sample). Once the cells adhered, they were irradiated with X-rays. At 0, 24, 48, and 72 h post-irradiation, 10 μL CCK-8 reagent (Med Chem Express, Princeton, NJ, USA) was added to each well and incubated for two h at 37°C. The absorbance was then measured at 450 nm with a Multiskan ascent (Thermo, Shanghai, China). The experiment was repeated three times.

### Colony formation assay

ESCC cells were seeded into 6-well plates and then irradiated at doses of 0, 2, 4, 6, 8, or 10 Gy. After 14 days of culture, the cells were fixed with 4% paraformaldehyde and stained with 0.5% crystal violet. Colonies with more than 50 cells were counted. To calculate survival curves (SF), the multitarget single-hit model was fitted to the data using the formula: [SF = 1 - (1 - e^-D/D0^)^N^].

### Wound-healing assay

The ESCC cells were seeded in 6-well plates. Cells in the 6 Gy groups were irradiated with X-ray, and the 0 Gy groups were not treated. At 90% confluence, a linear scratch wound was generated with a 200 µL pipette tip. After three rinses in PBS, a medium consisting of 1% FBS was added. The images were captured at 0 h, 24 h, and 48 h under a microscope (Nikon, Tokyo, Japan). All data were analyzed using Image J software.

### Flow cytometry analysis

Forty-eight h after irradiation, the transfected cells were harvested. Cell cycle distribution was determined via flow cytometry. Propidium iodide (MultiSciences Biotech Co., Ltd., Hangzhou, China) was used to stain cells for cell cycle detection.

### Statistical analysis

Non-operated patients diagnosed with stage I-III ESCC from January 2008 to December 2013 were enrolled in this study. Statistical analyses were performed using R software (Version 4.0.3) or GraphPad Prism software 5. We performed Kaplan-Meier (KM) survival analysis to evaluate 1-, 3-, 5-, and 10-year survival among ESCC patients with HMGB1-positive and HMGB1-negative expression and obtained* P* values. The different levels between the two groups of variables were evaluated by Fisher's exact test. All experimental data were repeated at least three times, and the experimental data were expressed as mean ± standard error. Comparisons of continuous variables between groups were performed by ANOVA. Statistical differences at *P*<0.05 were considered significant.

## Results

### Negative HMGB1 expression correlates with a long-term survival benefit in ESCC patients

**Figure 1:**
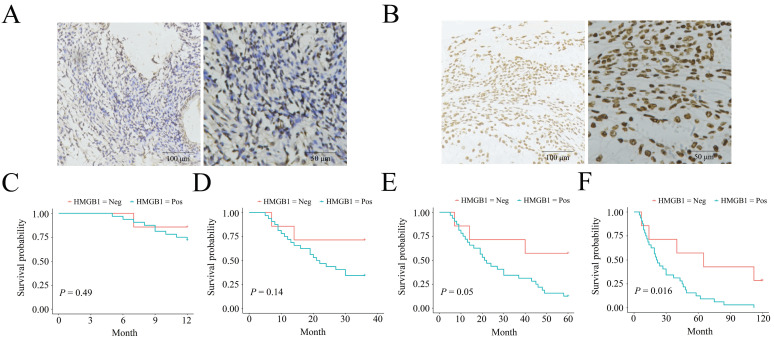
** HMGB1 expression status in ESCC biopsy specimens and the expression correlated with the survival. A.** Negative HMGB1 expression; B. Positive HMGB1 expression; One-y (**C**), three-y (**D**), five-y (**E**), and ten-y (**F**) survival among HMGB1-negative and HMGB1-positive groups according to Kaplan-Meier survival analysis.

The negative and positive HMGB1 expression profiles in ESCC are listed in Figure 1 A-B. HMGB1 expression was associated with clinical outcomes in ESCC patients. Patient data, including HMGB1 expression status, sex, age, lesion location, lesion length, gross tumor volume (GTV), T stage, N stage, and TNM stage, are shown in Table 1. The two groups had no statistical difference in terms of sex (*P*=0.686), age (*P*=1), tumor location (*P*=0.092), lesion length (*P*=0.683), GTV (*P*=0.092), T stage (*P*=0.075), and N stage (*P*=0.686), while the two groups statistically differed in the term of TNM stage (*P*=0.01) between the two groups. IHC confirmed the HMGB1 expression status in each ESCC tissue sample. KM survival analysis indicated no statistical differences in 1-, 3‐, and 5-year survival (*P*=0.49, *P*=0.14, and *P*=0.05, respectively). However, HMGB1 positive expression was negatively associated with 10-year survival of the patients (*P*=0.016), which suggested that positive HMGB1 expression correlated with poor long-term survival of ESCC patients (Figure 1C-F).

### Changes in HMGB1 localization after irradiation and the validation of HMGB1 overexpression

**Figure 2:**
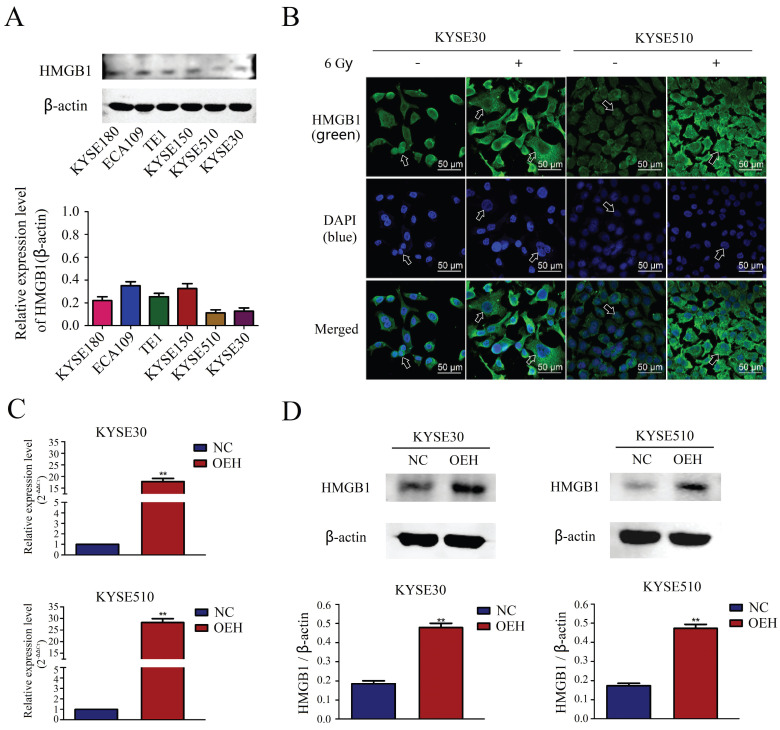
** The expression of HMGB1 in ESCC cell lines and the validation of HMGB1 overexpression efficiency. A.** The comparison of the HMGB1 protein expression levels in six esophageal cancer cell lines was performed using Western blotting; **B.** Immunofluorescence checking was shown and verified the specific localization of HMGB1 before and after irradiation. Green fluorescence, HMGB1 staining; blue fluorescence, nucleus staining; **C.** The mRNA expression level of HMGB1 was higher in OEH groups than that in NC groups; **D.** Overexpression of HMGB1 was validated using Western blotting. OEH, overexpression of HMGB1; NC, negative control.

The expression of HMGB1 in the KYSE180, ECA109, TE1, KYSE150, KYSE510, and KYSE30 ESCC lines was determined *in vitro* by Western blotting, and the results revealed lower expression levels of HMGB1 in the KYSE30, and KYSE510 cell lines compared with the other cell lines (Figure 2A). Therefore, we selected KYSE30 and KYSE510 cells for subsequent experiments. Using the laser confocal microscopy imaging, we investigated the cell-specific localization of HMGB1 in KYSE30 and KYSE510 cells, with or without 6 Gy X-ray treatment. As shown in Figure 2B, the results suggested that compared with the non-irradiated groups, the 6 Gy irradiated cells had an increased distribution of HMGB1 protein in the cytoplasm. The ESCC cell lines KYSE30 and KYSE510 stably overexpressing HMGB1 were successfully constructed by lentiviral transfection. RT-qPCR and Western blotting validated the transfection efficiency, and the validation results for all groups are shown in Figure 2C-D.

### HMGB1 overexpression significantly promoted proliferation and radioresistance of ESCC cells

**Figure 3:**
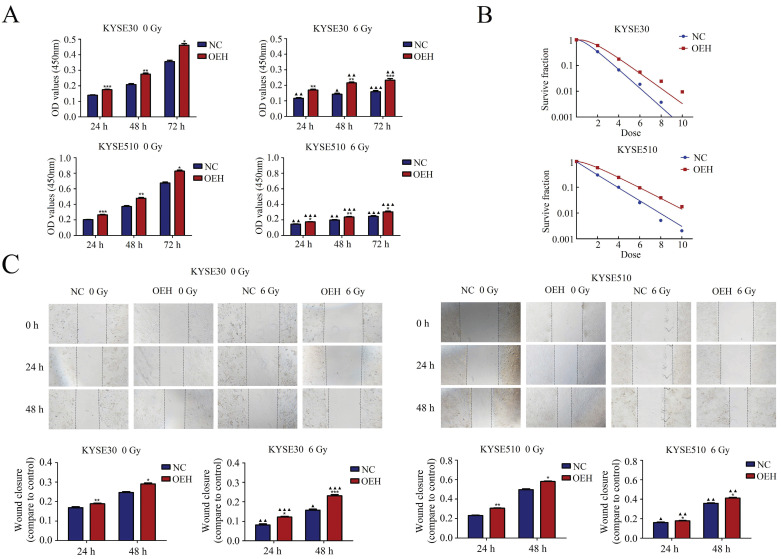
** HMGB1 protein overexpression increases the proliferation radioresistance and migration of ESCC cells. A.** Cell proliferation was detected by the CCK-8 assay; B. Colony formation assay was used to evaluate the radioresistance of KYSE30 and KYSE510 cells;** C.** Migration was detected by wound healing assays (magnification: 40×). The wound closure images were obtained at 0 h, 24 h, and 48 h. The migration capacities of irradiated groups at 24 h and 48 h were compared to the corresponding non-irradiated groups. Each experiment was replicated three times. The comparison with NC group is indicated by asterisks (*P<0.05, **P<0.01 and ***P<0.001), and the comparison with the corresponding non-irradiated group is indicated by triangles (▲*P*<0.05, ▲▲*P*<0.01 and ▲▲▲*P*<0.001). OEH, overexpression of HMGB1; NC, negative control.

As shown in Figure 3A, the CCK‐8 assay results showed that the cells overexpressing HMGB1 had significantly higher cell proliferation rates than the NC groups with or without irradiation (*P*<0.05). The rest of the groups showed decreased cell proliferation after irradiation, except for the KYSE30 overexpression HMGB1 group at 24 h, which showed no statistical difference after irradiation compared to its pre-irradiation counterpart (*P*>0.05). Subsequently, the effect of HMGB1 overexpression on the radioresistance of these cell lines was detected by colony formation assay (Figure 3B). The sensitizer enhancement ratios (SERs) were calculated by a reported method 27. SER = D0 (HMGB1 overexpression group)/D0 (NC group). Compared with NC groups, the survival curves of KYSE30 and KYSE510 HMGB1 overexpression groups were significantly changed with SERs 0.80 and 0.78. These results suggest that HMGB1 overexpression can promote ESCC cell proliferation and radioresistance.

### HMGB1 overexpression enhanced migration of ESCC cells

Cell migration was detected with the use of a wound-healing assay. The cells of HMGB1 overexpression indicated more rapid closure of the wound, which was remarkably different from that in NC cells (*P*<0.5), indicating increased cell motility. A similar trend was observed after exposure to 6 Gy X-ray in ESCC cells between the NC and the HMGB1 overexpression groups (*P*<0.5). The results, shown in Figure 3C, suggested that HMGB1 overexpression enhanced the migration of KYSE30 and KYSE510 cells.

### Effects of HMGB1 overexpression on cell cycle distribution

**Figure 4:**
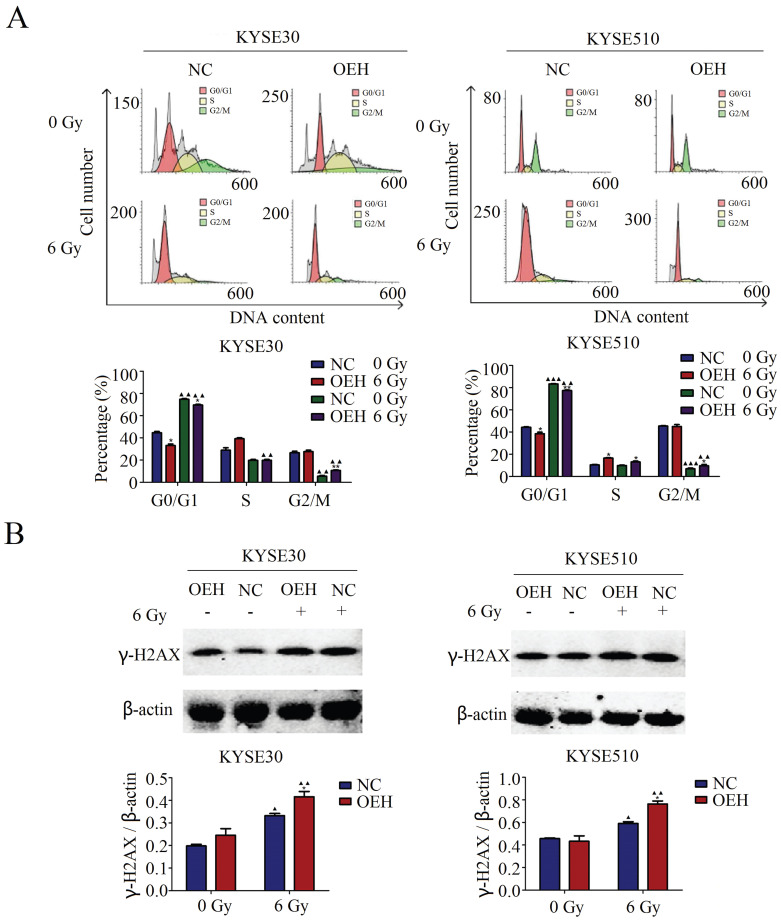
** HMGB1 protein overexpression regulated cell cycle distribution of ESCC cells. A.** HMGB1 alleviated the G0/G1 arrest of KYSE30 and KYSE510 after irradiation; **B.** There was a correlation between HMGB1 and γ-H2AX proteins after 6 Gy X-ray irradiation. Western blotting was used to detect the expression levels of γ-H2AX in KYSE30 and KYSE510 cells exposed or not to irradiation. Each experiment was replicated three times. The comparison with NC group is indicated by asterisks (*P<0.05), and the comparison with the corresponding non-irradiated group is indicated by triangles (▲*P*<0.05, *P*<0.01 and ▲▲▲*P*<0.001). OEH, overexpression of HMGB1; NC, negative control.

The results of the cell cycle distribution by flow cytometry suggested that the proportion of ESCC cells in the G0/G1 phase was significantly lower in the HMGB1 overexpression groups than in the control groups without 6 Gy X-ray irradiation (*P*<0.05). Irradiation caused cell cycle arrest at the G0/G1 phase in both control and HMGB1, overexpressing KYSE30 and KYSE510 cells (Figure 4A). Although irradiation arrested the ESCC cells at the G0/G1 phase in all groups, the HMGB1 overexpressing cells showed a weaker ability in G0/G1 phase arrest compared with the NC groups (*P*<0.05).

### HMGB1 affects the expression level of γ-H2AX after irradiation

γ-H2AX is the DNA damage marker and key signal for initiating DNA damage repair 28. It has been reported that in neurons, HMGB1 knockdown led to a weakened DNA damage response, which was reflected in the decreased expression of γ-H2AX 29. Therefore, we performed western blotting to confirm the relationship between HMGB1 and γ-H2AX in ESCC cells. As revealed in Figure 4B, the protein expression levels of γ-H2AX were examined in both the HMGB1 overexpression and NC groups with or without exposure to 6 Gy irradiation. Interestingly, all groups showed a significant enhancement in γ-H2AX expression levels after 6 Gy irradiation. In addition, γ-H2AX protein expression was higher in HMGB1 overexpressing cells than in the control groups (*P*<0.05).

## Discussion

EC is one of the most common malignant tumors in the digestive tract. Comprehensive treatment, mainly with radiotherapy, is the standard nonsurgical therapy for EC patients 30. Despite the significant progress in ESCC diagnosis and treatment, the 5-year survival rate of ESCC patients is still low 31. Local recurrence was the most common reason for treatment failure from radioresistance 32. Therefore, it is necessary to identify ESCC patients with resistance to radiotherapy and adjust their treatment plans accordingly to improve the radiotherapy efficacy.

HMGB1 is a DNA‐binding protein released during radiotherapy, and it is related to tumor radioresistance based on its DNA damage repair and autophagy functions 11. HMGB1 is involved in mechanisms that regulate resistance to radiotherapy in pancreatic 13, 14, cervical 8, breast 33, bladder 16, 34, and lung cancers 15.

In addition, Ma et al. found that HMGB1 expression was associated with ESCC recurrence after postoperative radiotherapy. Knocking down HMGB1 expression in ESCC cells could increase the radiosensitivity of these cells both *in vitro* and *in vivo*. Interestingly, HMGB1 could also inhibit the reduced autophagy levels in these cells, and activating autophagy restored the radioresistance 21. A similar finding was reported previously, where inhibiting HMGB1 could significantly increase the radiosensitivity of ESCC cells through G0/G1 phase arrest. Additionally, HMGB1 negative expression was correlated with the long-term survival of ESCC patients who received radiochemotherapy 24. Unlike previous reports, we selected ESCC cell lines with relatively low HMGB1 expression for overexpression in the present study. We verified the changes in HMGB1 protein localization in KYSE30 and KYSE510 cells before and after irradiation by immunofluorescence laser confocal technique. Because radiotherapy is an important treatment for ESCC, patients who had only received radiotherapy were included in this study for survival analysis to clarify the correlation between HMGB1 expression levels and survival of ESCC patients after radiotherapy alone.

We included 39 stage I-III ESCC patients who received radiotherapy in the present study. HMGB1 expression levels were associated with the 10-year survival rates of these patients, but no correlation was found in the 1-, 3-, and 5-year survival data.

This study explored the relationship between HMGB1 and ESCC radiosensitivity from a new perspective of HMGB1 overexpression. We observed that the overexpression of HMGB1 could enhance the migration, proliferation, and radioresistance of ESCC cells before and after radiation treatment *in vitro*, and HMGB1 may play an important role in the occurrence and development of EC. Amornsupak et al. believed that tumor cytoplasmic levels of HMGB1 could be used as an independent prognostic indicator for metastasis or recurrence in breast cancer patients 35. Li et al. reported that overexpression of HMGB1 can facilitate cervical cell migration by IHC analysis of cervical cancer tissues from 239 patients 36.

Shi et al. provided evidence supporting a close correlation between HMGB1 overexpression and gallbladder cancer progression and poor prognosis 37. Moreover, Dong et al. suggested that HMGB1 was associated with the 5-year overall survival of gastric cancer patients after analyzing the survival data of 100 patients with stage IIIA gastric cancer 38.

γ-H2AX plays a critical role in forming chromatin-remodeling complexes, and it also participates in the DNA damage and repair work associated with radiotherapy 39. γ-H2AX is closely related to DNA repair in tumor cells after radiotherapy. It is involved in radiotherapy-related DNA damage and repair in many cancers, including colorectal cancer 40, hepatocellular carcinoma 41, and others. Moreover, according to Kawashima et al., after colorectal cancer cells were irradiated, their γ-H2AX expression levels increased, and cell viability also decreased time-dependent 40. We found that ESCC cells overexpressing HMGB1 had higher γ-H2AX levels than the NC groups with 6 Gy irradiation.

Moreover, it has been confirmed that radiotherapy could induce a G0/G1 cell cycle arrest and pro-apoptotic effect in EC 42, 43, nasopharyngeal carcinoma 44, meningiomas 45, and breast cancer 46. Our results corroborate these findings, which revealed that KYSE30 and KYSE510 cells showed cell cycle arrest at the G0/G1 phase after radiotherapy, and this cell cycle arrest was weakened after overexpressed HMGB1. The limitation of the article is that validation of normal esophageal cells and animal experiments were not performed. Follow-up external validation and in-depth studies are still necessary.

## Conclusion

In this study, we demonstrated that positive HMGB1 expression in ESCC tissues was negatively correlated with 10-year survival rates of patients. Overexpressing HMGB1 promoted the migration, proliferation, and radioresistance of ESCC cells by reducing G0/G1 phase arrest. In conclusion, this study provides a theoretical basis that HMGB1 may be a potential target for overcoming ESCC radioresistance.

## Figures and Tables

**Table 1:** The relationship between HMGB1 protein expression and clinicopathological characteristics of ESCC patients

Variables	Overall	HMGB1			*P* value
	(n = 39)	Negative (n = 7)		Positive (n = 32)	
Sex					0.686
Female	15 (38.46%)	2 (5.13%)		13 (33.33%)	
Male	24 (61.54%)	5 (12.82%)		19 (48.72%)	
Age					1
< 70-year	18 (46.15%)	3 (7.69%)		15 (38.46%)	
≥ 70-year	21 (53.85%)	4 (10.26%)		17 (43.59%)	
Lesion location					0.092
Cervical-Upper	16 (41.03%)	6 (15.38%)		14 (35.9%)	
Middle-Lower	23 (58.97%)	1 (2.56%)		18 (46.15%)	
Lesion length					0.683
< 5 cm	18 (46.15%)	4 (10.26%)		14 (35.9%)	
≥ 5 cm	21 (53.85%)	3 (7.69%)		18 (46.15%)	
GTV					0.092
< 30 cm^3^	20 (51.28%)	6 (15.38%)		14 (35.9%)	
≥ 30 cm^3^	19 (48.72%)	1 (2.56%)		18 (46.15%)	
T stage^a^					0.075
T1-2	14 (35.9%)	5 (12.82%)		9 (23.08%)	
T3-4	25 (64.1%)	2 (5.13%)		23 (58.97%)	
N stage^a^					0.686
N0	15 (38.46%)	2 (5.13%)		13 (33.33%)	
N1-2	24 (61.54%)	5 (12.82%)		19 (48.72%)	
TNM stage^a^					0.01
I	8 (20.51%)	2 (5.13)		6(15.38%)	
II	13 (33.33%)	5 (12.82%)		8 (20.51%)	
III	18 (46.15%)	0 (0%)		18 (46.15%)	

^a^ The American Joint Committee on Cancer 6^th^ edition staging system was used to determine tumor stage for patients. HMGB1, high mobility group box 1; ESCC, esophageal squamous cell carcinoma; GTV, gross tumor volume.

## Abbreviations

HMGB1: high mobility group box 1
ESCC: esophageal squamous cell carcinoma
γ-H2A: phosphorylated histone H2AX
EC: esophageal cancer
DSBs: double-strand breaks
CSC: cancer stem cell
EMT: epithelial-mesenchymal transition
IHC: immunohistochemistry
FBS: fetal bovine serum
PBS: phosphate buffer saline
NC: negative control
RT-qPCR: real-time quantitative polymerase chain reaction
PVDF: polyvinylidene fluoride
TBST: TBS containing 0.1% Tween 20
SF: survival curves
KM: Kaplan-Meier
GTV: gross tumor volume
SERs: sensitizer enhancement ratios
